# Negative emotions associated with self-growth among older adults during the COVID-19 pandemic

**DOI:** 10.1192/j.eurpsy.2022.1271

**Published:** 2022-09-01

**Authors:** S. Von Humboldt, N.M. Mendoza-Ruvalcaba, E.D. Arias-Merino, J.A. Ribeiro-Gonçalves, E. Cabras, G. Low, I. Leal

**Affiliations:** 1 ISPA – Instituto Universitário, Lisbon, Portugal, William James Research Center, Lisbon, Portugal; 2 Universidad de Guadalajara CUTONALA, Health Sciences Division, Guadalajara, Mexico; 3 Universidad de Guadalajara CUCS, Public Health Department, Guadalajara, Mexico; 4 Universidad Antonio de Nebrija, Departamento De Educación, Madrid, Spain; 5 University of Alberta, Faculty Of Nursing, Edmonton, Canada

**Keywords:** Qualitative study, Covid-19 pandemic, Self-growth, Older Adults

## Abstract

**Introduction:**

The Covid-19 pandemic appeared globally (1), thus affecting the self-growth of the older population (2).

**Objectives:**

The aim of this study is to identify and analyze the negative emotions felt during the pandemic, as well as their impact on self-growth of 226 older individuals of four nationalities: Mexican, Italian, Portuguese and Spanish.

**Methods:**

Thus, a transnational qualitative survey was carried out. A content analysis was performed.

**Results:**

Seven negative emotions were reported, namely: fear, sadness, anger, grief, annoyance, loneliness and shame. These emotions were considerably associated with the following themes: (1) Sharing experiences; (2) Availability of the partner; (3) Spirituality and religion; (4) Be active; (5) Interest in new projects; (6) Civic participation; (7) Sexual activity. Older participants with Mexican and Italian nationality reported that sharing experiences as the most relevant topic, while for the Portuguese and Spanish participants, having a partner available was more important.

**Conclusions:**

This study demonstrated that negative emotions cooperated with the self-growth of older individuals during the Covid-19 pandemic. The heterogeneity of experiences lived by each culture was highlighted, underlining the positive side of negative emotions and their strong connection with the self-growth of the older people. 1.von Humboldt S et al. Smart technology and the meaning in life of older adults during the Covid-19 public health emergency period: A cross-cultural qualitative study. *Int Rev Psychiatry*, 2020; 1-10. 2. von Humboldt S et al. Does spirituality really matter? - A study on the potential of spirituality to older adult’s adjustment to aging. *Jpn Psychol Res, 56;*114-125.

**Disclosure:**

No significant relationships.

